# Anticancer Activity and Molecular Mechanisms of Acetylated and Methylated Quercetin in Human Breast Cancer Cells

**DOI:** 10.3390/molecules29102408

**Published:** 2024-05-20

**Authors:** Kozue Sakao, Shihomi Hamamoto, Daigo Urakawa, Ziyu He, De-Xing Hou

**Affiliations:** 1 The United Graduate School of Agriculture Sciences, Kagoshima University, Kagoshima 890-0065, Japan; k6044078@kadai.jp (D.U.); k7715393@kadai.jp (Z.H.); k8469751@kadai.jp (D.-X.H.); 2 Graduate School of Agriculture, Forestry and Fisheries, Kagoshima University, Kagoshima 890-0065, Japan

**Keywords:** quercetin, quercetin derivatives, methylation, acetylation, breast cancer, anticancer

## Abstract

Quercetin, a flavonoid polyphenol found in many plants, has garnered significant attention due to its potential cancer chemoprevention. Our previous studies have shown that acetyl modification of the hydroxyl group of quercetin altered its antitumor effects in HepG2 cells. However, the antitumor effect in other cancer cells with different gene mutants remains unknown. In this study, we investigated the antitumor effect of quercetin and its methylated derivative 3,3′,4′,7-*O*-tetramethylquercetin (4Me-Q) and acetylated derivative 3,3′,4′,7-*O*-tetraacetylquercetin (4Ac-Q) on two human breast cancer cells, MCF-7 (wt-p53, caspase-3-ve) and MDA-MB-231 (mt-p53, caspase-3+ve). The results demonstrated that 4Ac-Q exhibited significant cell proliferation inhibition and apoptosis induction in both MCF-7 and MDA-MB-231 cells. Conversely, methylation of quercetin was found to lose the activity. The human apoptosis antibody array revealed that 4Ac-Q might induce apoptosis in MCF-7 cells via a p53-dependent pathway, while in MDA-MB-231 cells, it was induced via a caspase-3-dependent pathway. Furthermore, an evaluation using a superoxide inhibitor, MnTBAP, revealed 4Ac-Q-induced apoptosis in MCF-7 cells in a superoxide-independent manner. These findings provide valuable insights into the potential of acetylated quercetin as a new approach in cancer chemoprevention and offer new avenues for health product development.

## 1. Introduction

Quercetin is a flavonoid polyphenol widely distributed in plants. Quercetin has attracted significant attention due to its antioxidant [1], anti-inflammatory [2], and related antitumor effects. However, quercetin’s potential as a cancer chemoprevention agent has been constrained by its low bioavailability due to low water solubility and rapid clearance in the body caused by fast metabolism and enzymatic degradation. Consequently, extensive research efforts have focused on modifying quercetin to develop analogs with potentially improved properties. Alkylation methods, such as methylation, which are relatively straightforward to synthesize, and acylation methods, such as acetylation, have been actively investigated as means of modulating quercetin’s bioactivity.

Methylation occurs in plants as well as in vivo to improve the bioavailability and stability of flavonoids. The methylation of flavonoids is carried out by the highly diverse *O*-methyltransferase (OMT) enzyme [3]. As a result, many types of methylated derivatives exist as natural compounds and have been isolated. Methylated flavonoids isolated from plants include compounds with quercetin as the basic skeleton, and isorhamnetin [4] and rhamnetin [5], known in vivo metabolites of quercetin, have also been identified. Rhamnadine, isolated from *Rhamnus petiolaris* [6], is a compound in which the hydroxy groups at sites 3 and 7 of quercetin are methylated and has been shown to reduce the cell proliferation and invasion of hepatocellular carcinoma cells [7]. The 3,7,4′-tri-*O*-methylated form of quercetin, known as ayanine, has been reported as a potent inhibitor of breast cancer resistance protein (BCRP) [8] and has recently shown promise in the treatment of *methicillin-resistant Staphylococcus aureus* infections [9]. Pachypodol is particularly noteworthy for its diverse biological activities, including anti-inflammatory, antioxidant, antimutagenic, and anticancer effects [10]. Furthermore, quercetin pentamethyl ether (QPE) is found in black turmeric (*Kaempferia parviflora* Wall. Ex Baker) and has been attracting attention in recent years [11,12,13,14]. This compound has a structure in which all five hydroxyl groups present in quercetin are methylated. QPE is reported to exhibit α-glucosidase inhibitory activity [15], anti-diabetic activity [14], and the stimulation of SIRT1 activity [16].

On the other hand, there are few reports on acylated derivatives of naturally occurring flavonoids, with most of them being compounds in which the sugar of the glycoside is acylated and modified [17]. Instead, the synthesis of acylated flavonoid derivatives has been actively studied due to the advantages of high yield and adequate evaluation of bioactivity. Quercetin acylated derivatives with desirable properties with potential anticancer applications have been obtained through various synthetic pathways. Quercetin aspirinate, 3-(7-*O*-acyl) quercetin aspirinate, 7-(3-*O*-acyl) quercetin aspirinate, and 3-quercetin aspirinate exhibited high cytotoxic activity against liver (HepG2) and promyelocytic leukemia (HL-60) cancer cells [18]. Pentaacetylquercetin, di(tetraacetylquinoyl)quercetin, and tri(diacetylcaffeoyl)quercetin showed the highest cytotoxicity against HeLa and NIH-3T3 cells [19]. Interestingly, all of these compounds were more effective than quercetin, irrespective of the cell type. Pentaacetylquercetin also inhibited the proliferation of MCF-7 cells, with an IC_50_ value that was two-thirds that of the parent compound, quercetin [20].

**Figure 1:**
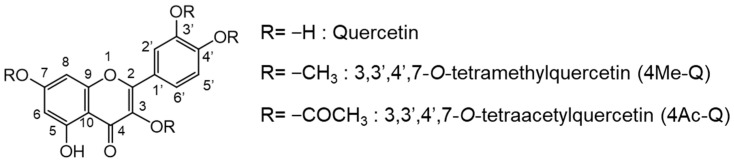
Structures of quercetin, 3,3′,4′,7-*O*-tetramethylquercetin (4Me-Q), and 3,3′,4′,7-*O*-tetraacetylquercetin (4Ac-Q).

We previously synthesized 3,3′,4′,7-*O*-tetraacetylquercetin (4Ac-Q) (Figure 1), which has been demonstrated to exhibit a higher inhibitory effect on cell proliferation and induce apoptosis compared with quercetin in HL-60 [21] and HepG2 cells [22]. Its induction of apoptosis was mediated by the caspase-3 pathway. Therefore, we aimed to assess its effect on cancer cells lacking caspase-3 activity and selected caspase-3-deficient MCF-7 cells [23,24] as one of the targets for evaluation in this study. While quercetin has been reported to demonstrate p53-dependent anticancer activity, it remains unclear whether its derivative, 4Ac-Q, also exhibits p53-dependent anticancer activity. Consequently, we also selected MDA-MB-231 cells, which are caspase-3 positive but p53-mutant, for evaluation alongside MCF-7 cells. In experiments with HL-60 cells, 4Ac-Q demonstrated an ROS-independent induction of apoptosis. Conversely, experiments with HepG2 cells treated with 4Ac-Q confirmed the generation of ROS within mitochondria during apoptosis induction. These results prompted an investigation into the correlation between 4Ac-Q-induced apoptosis and ROS levels in breast cancer cells, employing MnTBAP, a superoxide dismutase (SOD)-mimetic agent, in the present study. Our synthesized 3,3′,4′,7-*O*-tetramethylquercetin (4Me-Q) (Figure 1) was also evaluated in HL-60 cells and demonstrated lower anticancer activity compared with quercetin. However, retusin, a naturally occurring compound structurally identical to 4Me-Q, has been reported as a potent inhibitor of BCRP, like ayanine [8]. This suggests the potential activity of 4Me-Q against breast cancer in our study.

However, the antitumor effect of these modified quercetin derivatives in other cancer cells with different genes mutants remains unknown. In this study, we first examined the antitumor effects of quercetin and its methylated (4Me-Q) and acetylated (4Ac-Q) derivatives on two types of human breast cancer cells, MCF-7 (wt-p53, caspase-3-ve) and MDA-MB-231 (mt-p53, caspase-3+ve). Then, we used a human apoptosis antibody array to investigate their molecular actions, consequently investigating the superoxide independence by using a superoxide inhibitor, MnTBAP. Our data will become the first data to clarify the antitumor effects and molecular mechanisms of these modified quercetin derivatives on two human breast cancer cells, MCF-7 and MDA-MB-231, with different genes mutants.

## 2. Results

### 2.1. Comparative Analysis of the Inhibitory Effects of Quercetin, 4Me-Q, and 4Ac-Q on Cell Proliferation

#### 2.1.1. MTS Assay

**Figure 2:**
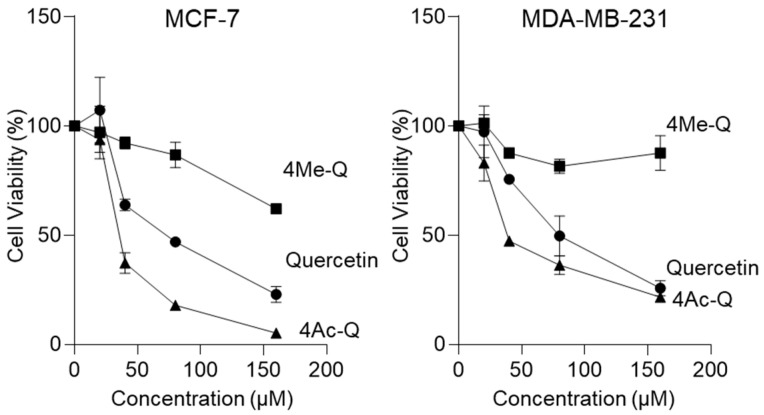
The effect of quercetin, 4Me-Q, and 4Ac-Q on the proliferation of MCF-7 and MDA-MB-231 cells. The cells were placed into a 96-well plate and treated with different concentrations of each compound or 0.1% DMSO (control) for 48 h. The cell density was assessed colorimetrically after staining with MTS and expressed as the optical density ratio of the treatment to the control at 499 nm. The data shown represent the means ± SD of three or more independent experiments.

Initially, we used MCF-7 and MDA-MB-231 cells to compare the effect of quercetin, 4Me-Q, and 4Ac-Q on the proliferation at multiple concentrations. As depicted in Figure 2, both quercetin and 4Ac-Q exhibited a concentration-dependent inhibition of proliferation in MCF-7 and MDA-MB-231 cells. Furthermore, both compounds demonstrated high cytotoxicity in MCF-7 cells compared with MDA-MB-231 cells. Specifically, the IC_50_ value of 4Ac-Q in MCF-7 cells was notably low at 37 μM, which is less than half of quercetin’s IC_50_ value (73 μM). Significant cytotoxic effects were observed in MDA-MB-231 cells at 48 μM, representing half the IC_50_ value of quercetin (85 μM) (Table 1). On the other hand, 4Me-Q was cytotoxic to both MCF-7 and MDA-MB-231 cells, but no significant proliferation inhibition was observed, even at 160 μM treatment.

#### 2.1.2. Colony Forming Assay

**Figure 3:**
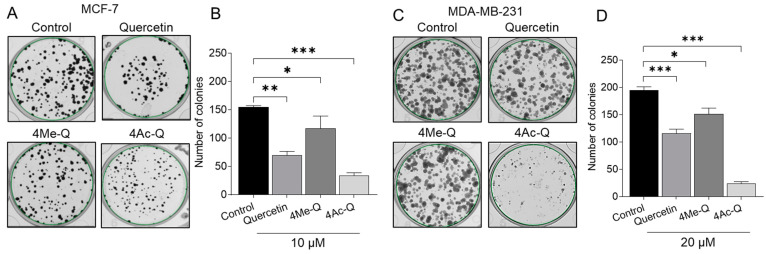
Effect of quercetin, 4Me-Q, and 4Ac-Q on the colony formation of MCF-7 and MDA-MB-231 cells. (**A**,**C**) Representative images of MCF-7 and MDA-MB-231 breast cancer cell colonies treated with indicated compounds. (**B**,**D**) The quantitative analyses of colony numbers of MCF-7 and MDA-MB-231 cells. The cells were placed into a 6-well plate and treated with indicated concentrations of each compound or 0.1% DMSO (control) for 9 days. The data shown represent the means ± SD of three or more independent experiments. * mark denoted significant differences (* *p* < 0.05, ** *p* < 0.01, *** *p* < 0.001) between the control and Q, 4Me-Q, or 4Ac-Q.

We then evaluated the effects of long-term treatment with lower concentrations of quercetin, 4Me-Q, and 4Ac-Q on the proliferative potential of MCF-7 and MDA-MB-231 cells using a colony formation assay. In Figure 3A,C, a significant inhibition of colony formation was observed in MCF-7 cells when treated with 10 μM samples for 9 days and in MDA-MB-231 cells when treated with 20 μM samples for 9 days. Notably, 4Ac-Q exhibited a stronger inhibition of colony formation than quercetin, with a 2-fold inhibition in MCF-7 cells (Figure 3B) and more than a 5-fold inhibition in MDA-MB231 cells (Figure 3D). Interestingly, a markedly higher inhibitory effect on both cell lines was also observed with 4Me-Q. To evaluate differences in cellular response to quercetin, 4Me-Q, and 4Ac-Q treatment, additional studies were performed at a constant concentration of 40 µM for both cell lines.

### 2.2. Apoptosis-Inducing Ability of Quercetin Is Enhanced by Acetylation and Attenuated by Methylation

#### 2.2.1. Detection of Cell Membrane Changes Using Annexin V/PI

We previously reported that 4Ac-Q induced apoptosis in the human promyelocytic leukemia cell line (HL-60) and in HepG2 human liver cancer cells more potently than quercetin [21,22]. In HL-60 cells, we also reported that methylation of the hydroxy group of quercetin lost its ability to induce apoptosis. In this study, apoptosis-inducing processes were assessed by quantitative measurements using annexin-V/PI staining, the release of histone-related DNA fragments into the cytoplasm, and JC-1 dye for monitoring mitochondrial membrane potential changes.

**Figure 4:**
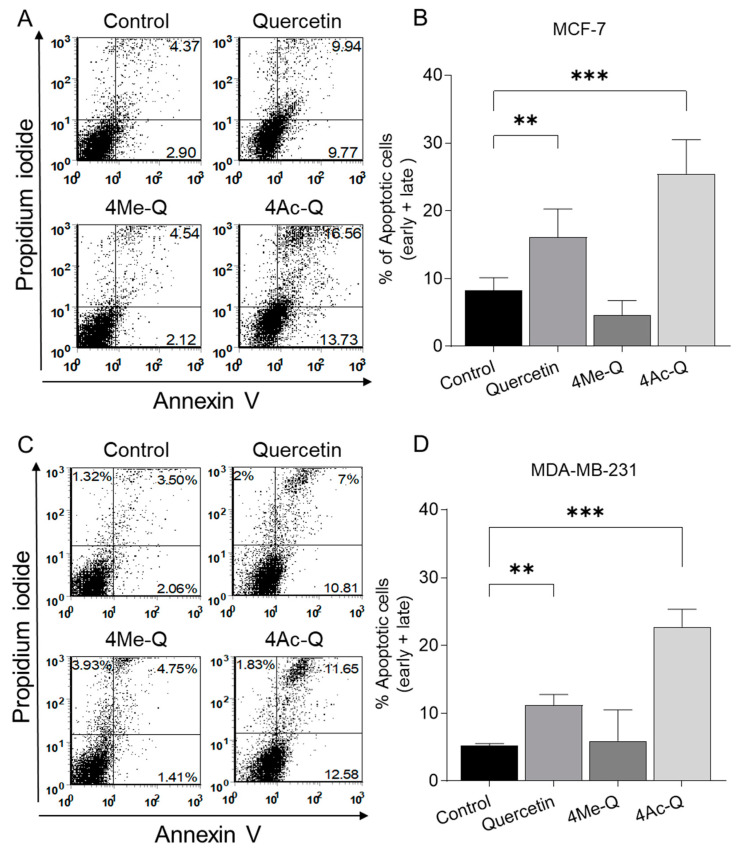
Effect of quercetin, 4Me-Q, and 4Ac-Q on the apoptosis induction in MCF-7 and MDA-MB-231 cells. Representative flow histograms depicting the apoptotic fraction in (**A**) MCF-7 and (**C**) MDA-MB-231 cells. Quantitation of % apoptotic fraction (early + late apoptotic cells) in (**B**) MCF-7 and (**D**) MDA-MB-231 cells. Treated for 48 h with DMSO (control) or the indicated concentrations of quercetin, 4Me-Q, and 4Ac-Q. Data represented as the means ± SD; * mark denotes significant differences (** *p* < 0.01, *** *p* < 0.001) between the control and each sample.

Figure 4A,C shows a representative flow histogram of annexin V/PI fluorescence in each cell after treatment with 40 µM quercetin, 4Me-Q, 4Ac-Q, or DMSO (control) for 48 h. Treatment with quercetin or 4Ac-Q induced significant apoptosis in MCF-7 and MDA-MB-231 cells, whereas 4Me-Q did not. In MCF-7 cells, quercetin treatment induced 16.1% apoptosis, while 4Ac-Q treatment induced 25.4% apoptosis. For MDA-MB-231 cells, quercetin and 4Ac-Q treatments induced 11.2% and 22.7% apoptosis, respectively.

#### 2.2.2. DNA Fragmentation and Western Blotting Analysis of PARP Cleavage

**Figure 5:**
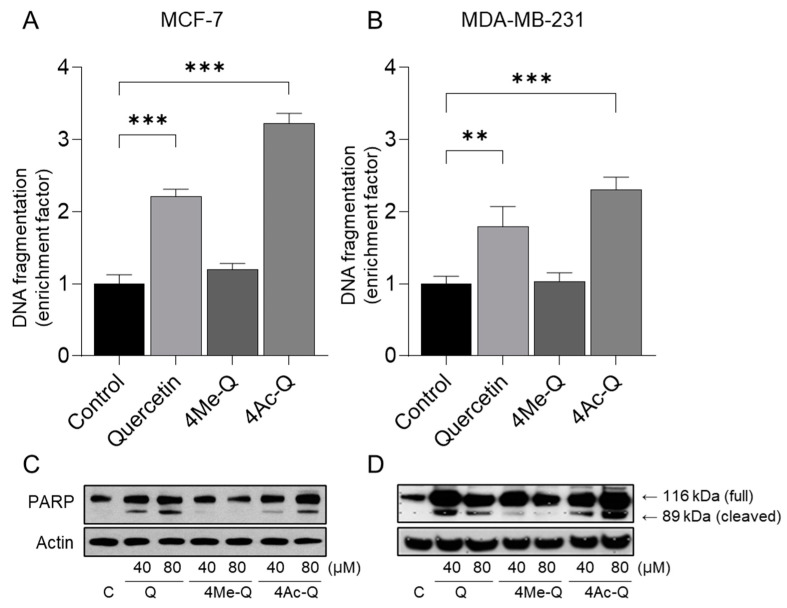
Quantification of apoptotic DNA fragments in (**A**) MCF-7 cells and (**B**) MDA-MB-231 cells treated with quercetin, 4Me-Q, and 4Ac-Q. Cells were grown in the absence (control) or the presence of compounds (40 μM) for 48 h. The percentages of cells with hypodiploid DNA contents represent the fractions undergoing apoptotic DNA. (**C**,**D**) Immunoblot of PARP cleavage. Cellular lysate was applied on 10% of SDS-PAGE. PARP was detected with the corresponding specific antibodies and visualized by a chemiluminescence ECL kit. Data are represented as the means ± SD. ** *p* < 0.01, *** *p* < 0.001 compared with the untreated group.

The cleavage of genomic DNA into histone-associated DNA fragments, a biochemical indicator of cellular apoptosis, was validated using an ELISA kit. Quercetin and 4Ac-Q treatment resulted in a statistically significant increase in histone-associated DNA fragment release into the cytosol (a measure of apoptosis) over the DMSO-treated control in MCF-7 and MDA-MB-231 cells (Figure 5A,B). 4Me-Q treatment failed to increase the release of histone-associated DNA fragments into the cytosol over the DMSO-treated control in MCF-7 and MDA-MB-231 cells.

We then confirmed the cleavage of PARP as one possible reason for histone-related DNA fragmentation by quercetin or 4Ac-Q using Western blotting. As shown in Figure 5C,D, cleavage of PARP was confirmed in MCF-7 and MDA-MB-231 cells through quercetin and 4Ac-Q treatment. In particular, cleavage of PARP was more intensely detected in MDA-MB-231 cells than in MCF-7 cells, which are deficient in caspase-3.

#### 2.2.3. Measurement of Mitochondrial Membrane Potential Changes during Apoptosis

An early prominent feature of programmed cell death is the destruction of active mitochondria, including changes in membrane potential and redox potential. In some apoptosis systems, the loss of the mitochondrial membrane potential may serve as an initial event in the apoptosis process [25]. JC-1 dye can be utilized as an early marker of apoptosis, indicating changes in the mitochondrial membrane potential. We therefore used quercetin, 4Me-Q, and 4Ac-Q to examine the effects of each compound on mitochondrial membrane potential in MCF-7 and MDA-MB-231 cells after 4 h of treatment.

**Figure 6:**
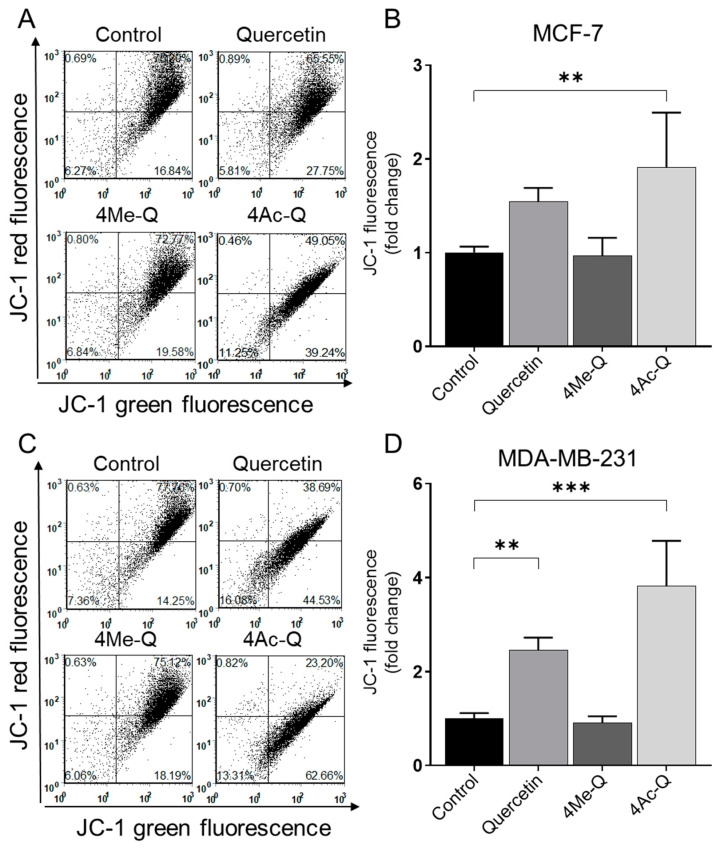
Flow cytometric quantitation of JC-1 fluorescence (a measure of mitochondrial membrane potential) in (**A**) MCF-7 and (**C**) MDA-MB-231 cells after 4 h of treatment with DMSO (control) or the 40 μM concentrations of quercetin, 4Me-Q, and 4Ac-Q. Quantitation of monomeric (green) JC-1 fluorescence in (**B**) MCF-7 and (**D**) MDA-MB-231 cells. Each experiment was performed at least three times, and representative data from one such experiment is shown. For quantitative measurements, the results are expressed as the fold change to each control (means ± SD, *n* = 3). * mark in (**B**,**D**) denoted significant differences (** *p* < 0.01, *** *p* < 0.001) between the control and each compound.

Consistent with the results of the present study on other apoptosis-inducing measurements, both MCF-7 and MDA-MB-231 cells showed disruption of mitochondrial membrane potential upon treatment with quercetin or 4Ac-Q, as indicated by the flow cytometric analysis of monomeric (green) JC-1 fluorescence (Figure 6). Interestingly, sensitivity to membrane potential changes was greater in MDA-MB-231 cells than in MCF-7 cells, as evidenced by the fact that quercetin treatment significantly reduced membrane potential.

### 2.3. 4Ac-Q Shows ROS-Independent Inhibition of Cell Proliferation in MCF-7 Cells

**Figure 7:**
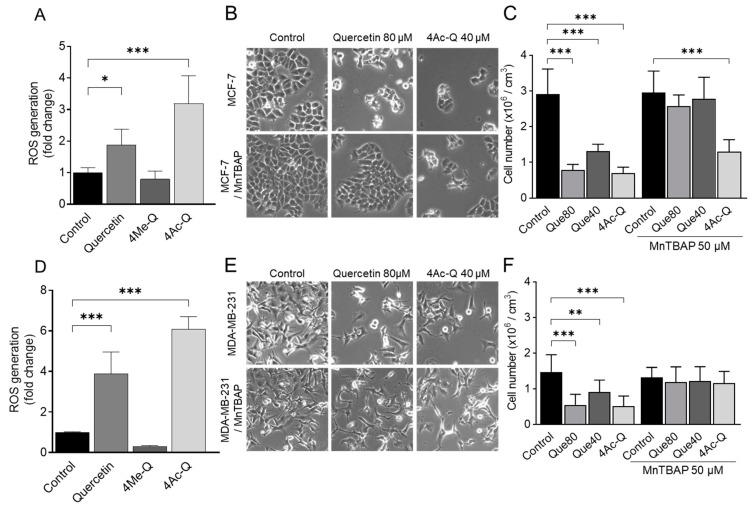
Effect of quercetin, 4Ac-Q, and MnTBAP on mitochondrial ROS production. (**A**,**D**) Flow cytometric quantitation of MitoSOX red fluorescence in MCF-7 and MDA-MB-231 cells after 30 min of treatment with 0.1% DMSO (control) or the indicated concentrations of quercetin, 4Me-Q, and 4Ac-Q. Results are expressed as the fold change of MitoSOX red fluorescence relative to control. (**B**,**E**) Morphological changes in MCF-7 cells and MDA-MB-231 cells treated with quercetin or 4Ac-Q by MnTBAP treatment. (**C**,**F**) Number of the viability of MCF-7 cells and MDA-MB-231 cells treated with quercetin or 4Ac-Q by MnTBAP co-treatment. Both cells were pretreated with 50 μM MnTBAP for 1 h and then treated with 40 or 80 μM quercetin (Que40 or Que80) or 40 μM 4Ac-Q for another 48 h. The harvested cells were stained with trypan blue and analyzed using a cell counter. Data represented as the means ± SD. * mark denotes significant differences (* *p* < 0.05, ** *p* < 0.01, *** *p* < 0.001) between the control and each sample.

Based on the results depicted in Figure 6, the production of mitochondrial ROS, which is known to correlate with a decrease in mitochondrial membrane potential, was quantified using flow cytometry to assess the impact of quercetin, 4Me-Q, and 4Ac-Q on ROS production. As shown in Figure 7A,D, mitochondrial ROS production was confirmed at just 30 min after the addition of quercetin or 4Ac-Q. The ROS production by 4Ac-Q was 1.7-fold higher in MCF-7 cells and 1.5-fold higher in MDA-MB-231 cells. In contrast, ROS production by 4Me-Q supplementation was not observed in either cell.

**Figure A1:**
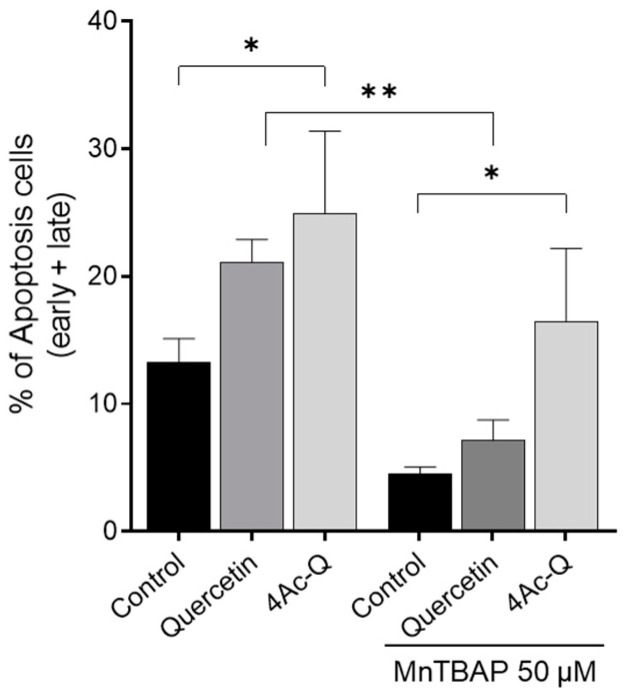
Effect of MnTBAP on quercetin and 4Ac-Q induced apoptosis. Flow cytometric quantitation of apoptosis in MCF-7 cells treated with quercetin or 4Ac-Q by MnTBAP co-treatment. Both of cells were pretreated with 50 μM MnTBAP for 1 h and then treated with 80 μM quercetin or 40 μM 4Ac-Q for another 48 h. * *p* < 0.05, ** *p* < 0.01 indicate significant differences between the control and each compound.

In our previous research, it was elucidated that quercetin and 4Ac-Q induced mitochondrial ROS production during apoptosis induction in HepG2 cells [22]. It was also clear that in HL-60 cells, quercetin-induced apoptosis was closely associated with the production of superoxide, while 4Ac-Q-induced apoptosis was not [21]. To clarify the differences in the role of mitochondrial ROS production between cell lines, we addressed this question using Mn (III) tetrakis (4-benzoic acid) porphyrin (MnTBAP) as a superoxide scavenger. MCF-7 cells and MDA-MB-231 cells were treated with quercetin and 4Ac-Q in the absence or presence of MnTBAP, and the proliferation inhibitory effect of each was determined by a trypan blue assay. Quercetin was treated at 80 µM, corresponding to the IC_50_ value for cell proliferation inhibition. As shown in Figure 7B,C, a significant recovery of proliferation inhibition was observed in MCF-7 cells with MnTBAP co-treatment compared with 80 µM quercetin alone (38.9% to 12.0%). However, this recovery was not observed between MnTBAP co-treatment and 40 µM 4Ac-Q alone (43.0% to 39.6%) (Figure 7C). Correspondingly, apoptosis induction by 4Ac-Q could also not be completely inhibited by the combination of MnTBAP (Figure A1). In contrast, both quercetin and 4Ac-Q showed reduced cell proliferation inhibition with MnTBAP co-treatment in MDA-MB231 cells (Figure 7E,F).

These results suggested that mitochondrial ROS production is important for the inhibition of MDA-MB-231 cell proliferation by quercetin and 4Ac-Q. In MCF-7 cells, comparable outcomes were achieved solely with quercetin treatment, suggesting that 4Ac-Q might trigger cell growth inhibition and apoptosis via a pathway that is not reliant on ROS production.

### 2.4. Differential Regulation of Apoptosis-Related Proteins by Quercetin and 4Ac-Q in MCF-7 and MDA-MB-231 Cells

The results of the MnTBAP experiments suggested that 4Ac-Q induces apoptosis in MCF-7 and MDA-MB-231 cells via distinct signaling pathways. Furthermore, the mechanisms of action for 4Ac-Q and quercetin may differ, potentially explaining the higher activity of 4Ac-Q compared with quercetin. Therefore, we compared differences in signaling pathways for apoptosis induction between cells and samples using the human apoptotic antibody array membrane.

**Figure 8:**
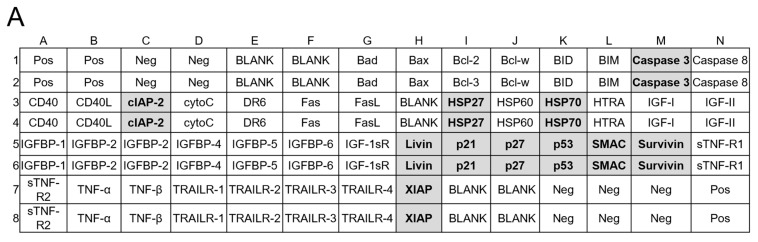

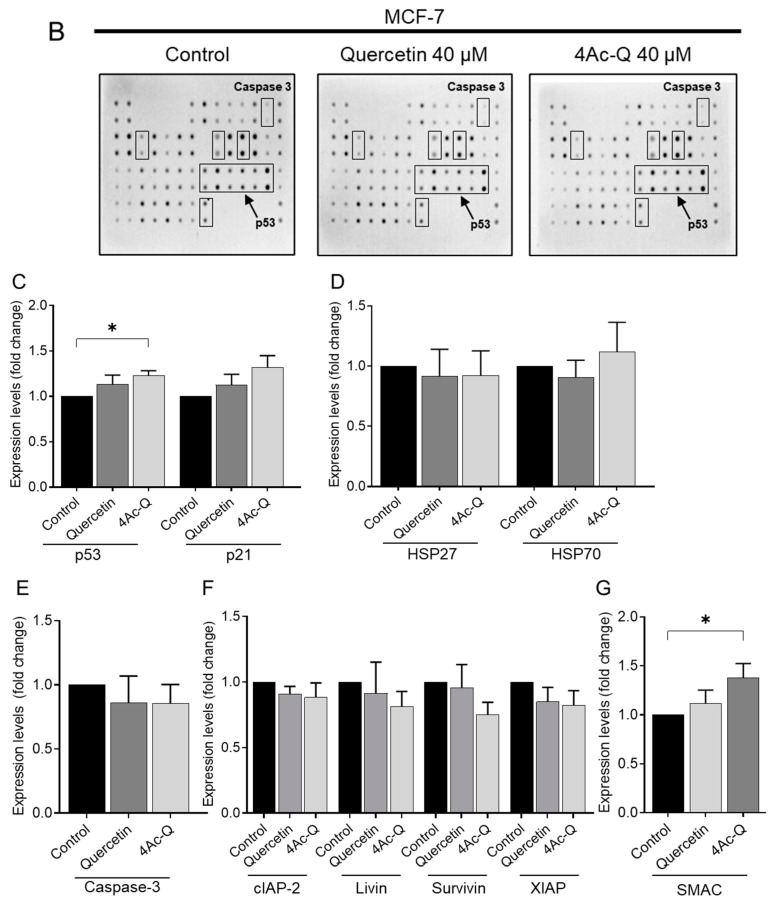

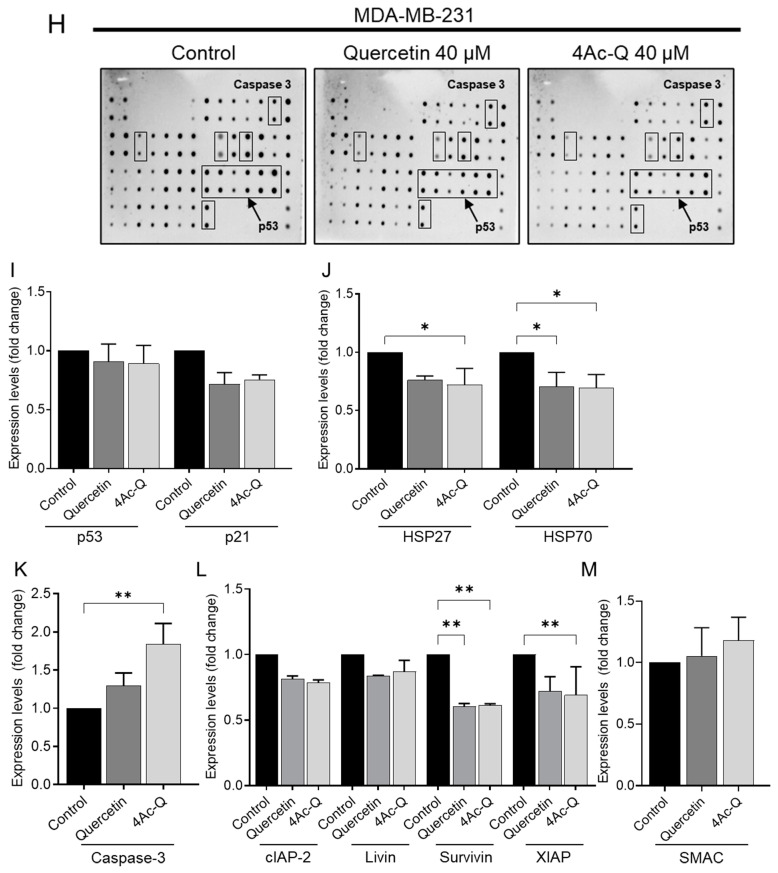
Human apoptotic antibody arrays in MCF-7 and MDA-MB-231 cells treated with quercetin and 4Ac-Q. (**A**) Array map of apoptosis-related protein on the human apoptosis antibody array membrane. Bold letters in the map indicate apoptosis-associated proteins graphed as C–G, I–M. (**B**,**H**) Array images indicating the protein levels of various apoptosis-related proteins in MCF-7 and MDA-MB-231 cells treated with 0.1% DMSO (control), 40 μM quercetin, or 4Ac-Q for 48 h. (**C**,**I**) The mean pixel density of p53 and p21; (**D**,**J**) HSP27 and HSP70; (**E**,**K**) cleaved caspase-3; (**F**,**L**) inhibitor of apoptosis proteins (IAPs), including cIAP-2, livin, surviving, and XIAP; and (**G**,**M**) IAP antagonist SMAC in the quercetin- or 4Ac-Q-treated group relative to the control. * *p* < 0.05, ** *p* < 0.01 indicate significant differences between the control and each compound.

As illustrated in Figure 8C, treatment with quercetin and 4Ac-Q resulted in the activation of p53 and a simultaneous increase in p21 levels in MCF-7 cells. Conversely, in p53-mutant MDA-MB-231 cells, p53 was marginally downregulated, followed by a decrease in p21 expression (Figure 8I). In MDA-MB-231 cells, the antiapoptotic proteins HSP27 and HSP70 were significantly downregulated by treatment with quercetin and 4Ac-Q (Figure 8J). In MCF-7 cells, quercetin slightly downregulated both proteins, while 4Ac-Q resulted in increased HSP70 levels (Figure 8D).

MCF-7 cells lacked caspase-3 and therefore showed no activity (Figure 8E), whereas in MDA-MB-231 cells, cleavage of the apoptotic protein caspase-3 was significantly detected in quercetin and 4Ac-Q treatments (Figure 8K). In both cell types, the expression of apoptosis inhibitory proteins such as cIAP-2, livin, survivin, and XIAP was downregulated by quercetin and 4Ac-Q, as depicted in Figure 8F,L. Notably, the inhibitory effect was more pronounced in MDA-MB-231 cells, particularly for survivin and XIAP, as evidenced in Figure 8L. Additionally, the expression level of SMAC, a mitochondrial pro-apoptotic protein crucial for apoptosis execution by IAP family inhibition [26], was elevated in both cell types (Figure 8G,M), with a higher increase observed in the 4Ac-Q treatment compared with quercetin treatment.

## 3. Discussion

### 3.1. Differential Impact of Quercetin, 4Me-Q, and 4Ac-Q on Cell Proliferation

An MTS assay demonstrated quercetin’s capacity to hinder cell proliferation aligned with prior findings, indicating that MDA-MB-231 cells exhibited less susceptibility to quercetin compared with MCF-7 cells [27,28,29]. Furthermore, quercetin exhibited a biphasic effect, promoting growth at low concentrations and exerting an antiproliferative influence at higher concentrations, as reported previously [30]. In contrast, 4Ac-Q displayed a suppressive impact on proliferation even at lower concentrations, demonstrating a superior inhibitory efficacy on cell proliferation compared with quercetin across both concentration ranges and cell types. The observed stimulation of cell proliferation by quercetin suggests a potential involvement of the estrogen receptor (ER) [31], implying that 4Ac-Q likely employs a distinct mechanism for growth inhibition compared with quercetin in ER-positive MCF-7 cells. On the other hand, 4Me-Q showed a lower inhibition of cell proliferation than quercetin, consistent with our MTT assay results using HL-60 cells [21]. Martins et al. compared the IC_50_ values of quercetin and 4Me-Q across various cancer cell lines, finding that 4Me-Q was consistently less cytotoxic than quercetin by a factor of three or less [32]. In particular, in MCF-7 cells, the IC_50_ of quercetin was 20.90 ± 3.44, with 4Me-Q > 100 [33], which is consistent with our results and supports the conclusion that methylation of the hydroxy group of quercetin reduces its inhibitory effect on cell proliferation. Interestingly, 4Me-Q notably inhibited colony formation in our study. Previous research by Landis-Piwowar suggested that methylated flavonoids induce G0/G1 cell cycle arrest rather than apoptosis in HL60 cells [20]. In line with their findings, the antiproliferative effect of 4Me-Q was not characterized by apoptosis in this study. Unlike the MTS assay, which gauges metabolic activity, the colony formation assay, which directly evaluates cell proliferation, may capture this specific inhibitory effect of 4Me-Q. This highlights the significance of employing multiple assays to comprehend how compounds like 4Me-Q impede cell proliferation.

Regarding quercetin’s inhibitory effect on cell proliferation, many reports indicate a marked effect in MCF-7 cells at 48 h treatment concentrations ranging from 20 µM to 100 µM [34,35]. Conversely, in MBA-MB-231 cells, while some studies indicate an IC_50_ value exceeding 100 μM [29,34], others report significant inhibition of cell proliferation from a concentration of 50 μM [27,36], as well as a cell viability of less than 50% after 48 h of treatment with 40 μM quercetin [37]. In our study, the IC_50_ values for quercetin treatment for 48 h were 73 μM for MCF-7 cells and 85 μM for MDA-MB-231 cells. Therefore, the primary use of a 40 µM treatment concentration in this study suggests that quercetin was assessed for anticancer activity at a moderate potency level.

### 3.2. Clarification and Comparison of Apoptosis-Inducing Pathways

In the present study, an increase in p21 was observed in p53 wild-type MCF-7 cells, along with an increase in p53, for both quercetin and 4Ac-Q; this trend was even stronger for 4Ac-Q. Conversely, p53 and p21 levels were decreased in p53-mutant MDA-MB-231 cells by both quercetin and 4Ac-Q. These findings are consistent with the report by Ranganathan et al., which indicated that p21 expression levels increased in quercetin-treated MCF-7 cells but decreased in MDA-MB-231 cells exposed to quercetin [38]. HSP27 and HSP70 were downregulated by quercetin treatment in MCF-7 and MDA-MB-231 cells, consistent with previous reports [29]. However, our results showed an exception with 4Ac-Q treatment in MCF-7 cells, where HSP70 exhibited a slight increase. According to Walerych et al., Hsp70 is required for p53 activation and the maintenance of its function [39,40]. It is possible that 4Ac-Q induces these phenomena by activating p53 levels more effectively or for a longer duration compared with quercetin.

The downregulation of IAP family proteins in MDA-MB-231 cells was consistent with other reports that quercetin downregulates XIAP and induces caspase-3-mediated apoptosis [41]. On the other hand, MCF-7 cells lacking caspase-3 also showed a downregulation of the IAP family, although it was not as pronounced. Apoptosis in MCF-7 cells deficient in caspase-3 has been reported to be mediated by other effector caspases such as caspase-6 and caspase-7 [42,43]. XIAP, c-IAP1, and c-IAP2 bind directly to activated caspase-3 and -7 and can inhibit their activity [44,45,46]. The downregulation of the IAP family by quercetin or 4Ac-Q may have led to increased activation of caspase-7 in MCF-7 cells and caspase-3 in MDA-MB-231 cells, thereby promoting apoptosis induction.

The behavior of proteins other than 4Ac-Q treatment in MCF-7 cells was in line with their respective cell characteristics, revealing that quercetin and 4Ac-Q may stimulate the same apoptotic pathway, especially in MDA-MB-231 cells.

### 3.3. Quercetin Induced Mitochondria ROS-Dependent Apoptosis

In Figure 7, 4Ac-Q suggested the existence of an apoptosis-inducing pathway in MCF-7 cells that is independent of mitochondrial ROS production. Liao et al. reported that apoptosis induced by *O*-alkylated derivatives of quercetin cannot be reversed by Z-VAD-FMK (a caspase inhibitor) or *N*-acetyl cysteine (an antioxidant), suggesting that this apoptosis is not dependent on caspases or ROS [47]. Our findings were consistent with their observations and indicated that acetyl substitution of the hydroxy group of quercetin could stimulate a pathway of apoptosis induction that is different from that of the parent compound. Apoptosis is governed by both endogenous pathways mediated by mitochondria-localized proteins and exogenous pathways mediated by death receptors (such as Fas and TNFR1), both of which involve the p53-mediated induction of apoptosis [48]. Thus, it is highly likely that 4Ac-Q mediates both pathways in p53 wild-type MCF-7 cells. The death receptor-mediated pathway involves external stimuli binding to receptors like the tumor necrosis factor (TNF) receptor and Fas receptor, resulting in apoptosis signal execution. This pathway is ROS-independent [49]. In this study, we utilized antibody arrays to simultaneously detect 43 human apoptosis markers, including relevant protein antibodies for both the endogenous and exogenous pathways. However, contrary to our expectations, we did not observe significant changes in the proteins associated with these exogenous pathways. A treatment time of 48 h may not have been suitable for accurately detecting changes in the expression of cell death receptor markers. Therefore, it is possible that the correct expression of these markers was not captured, emphasizing the need to explore different detection time points in future studies. Emphasis should be placed on investigating exogenous pathways to find novel signaling pathways that 4Ac-Q has.

### 3.4. Impact of Methylation and Acetylation of Hydroxy Groups of Quercetin on the Induction of Apoptosis

In this study, apoptosis was detected by detecting changes in plasma membrane structure, DNA fragmentation, and loss of mitochondrial membrane potential. For both MCF-7 and MDA-MB-231 cells, 4Ac-Q showed stronger ability to induce apoptosis than quercetin, and MCF-7 cells were more sensitive than MDA-MB-231 cells. 4Me-Q did not induce apoptosis in either cell. Landis-Piwowar et al. concluded that permethylated flavonoids are not potent cytotoxic to HL-60 cells [50], which is consistent with our previous report [21] and also supports the present results of 4Me-Q on breast cancer cells.

In our previous study, we elucidated the mechanism by which 4Ac-Q induces stronger apoptosis than quercetin in HepG2 cells [22]. We reported that acetyl modification of quercetin not only significantly increased its intracellular absorption but also enhanced its metabolic stability and prolonged its intracellular persistence, thereby increasing its capacity to induce apoptosis [22]. The hydroxy group of quercetin, when replaced by a methyl or acetyl group, decreases polarity and promotes cell membrane permeability. Nevertheless, the reason for the completely different ability of 4Me-Q and 4Ac-Q to induce apoptosis may be that methylation is more stable than acetylation in the medium or after intracellular uptake. Another possible reason for the failure to induce apoptosis could be the lack of mitochondrial ROS production after treatment with 4Me-Q (Figure 7A,D). The primary causes of this are believed to stem from decreased water solubility resulting from excessive methylation of the hydroxy groups, as well as diminished reactivity of the hydroxy groups due to substituents other than the functional groups.

The effect of 4Ac-Q on MDA-MB-231 cells is similar to that in HepG2 cells [22], showing a stronger effect than quercetin but occurring through the same pathway as quercetin, as shown in this study. Specifically, the increased activity of 4Ac-Q is believed to be based on the increase in cellular uptake, resulting in an elevated intracellular quercetin concentration and metabolic delay due to acetyl protection.

Although the effect of 4Ac-Q on MCF-7 cells is thought to be partly due to the same mechanism as quercetin, it also shows distinct behaviors: (a) inhibition of cell proliferation at low concentrations and (b) ROS-independent inhibition of cell proliferation and induction of apoptosis. This implies that 4Ac-Q has a signaling pathway that is partially distinct from quercetin. In the future, exploration of the structural potential of 4Ac-Q is required. Based on these findings, future studies necessitate a comprehensive evaluation utilizing standard drugs or their combination counterparts. Li et al. reported that doxorubicin, a chemotherapeutic agent used in breast cancer treatment, inhibited cell growth by approximately 70% at 1.0 μM in MCF-7 cells and approximately 60% at 1.5 μM in MDA-MB-231 cells. In comparison, quercetin alone demonstrated comparable efficacy at 60 μM [51]. In other words, there is around a 60-fold difference in anticancer activity between quercetin, a natural compound, and conventional anticancer drugs. In this study, 4Ac-Q enhanced the anticancer activity of quercetin by approximately 2-fold; however, it is difficult to utilize it alone as an anticancer drug due to its significantly lower efficacy compared with established treatments. Interestingly, the combinations of quercetin with anticancer agents have been reported to enhance the anticancer activity of anticancer agents [52,53,54,55]. For instance, Safira et al. reported that in MDA-MB-231 cells, the IC_50_ values of docetaxel and quercetin were 33 nM and 125 μM, respectively. Notably, the dose reduction index values showed that the IC_50_ dose of docetaxel could be reduced by 7-fold when used concomitantly with a combination of docetaxel (7 nM) and quercetin (95 μM) [52]. Therefore, further investigations should explore 4Ac-Q in combination with established anticancer agents to elucidate novel preventive effects and underlying mechanisms.

## 4. Materials and Methods

### 4.1. Chemicals, Antibodies, and Reagents

Quercetin for the synthesis of 4Ac-Q (purity ≥ 90%) was purchased from Sigma-Aldrich (St. Louis, MO, USA), and quercetin for the bioactivity assay (purity > 99%) was purchased from LKT Laboratories (St. Paul, MN, USA). 3-(4,5-dimethylthiazol-2-yl)-5-(3-carboxymethoxyphenyl)-2-(4-sulfophenyl)-2H-tetrazolium, inner salt (MTS) was purchased from Promega (Madison, WS, USA). An annexin V-FITC apoptosis detection kit was purchased from BD Biosciences (San Diego, CA, USA). 3,8-phenanthridine diamine-hexyl triphenylphosphonium iodide (MitoSOX red) and 5,5′,6,6′-tetrachloro-1,1′,3,3′-tetra-ethyl benzimidazolyl carbocyanine iodide (JC-1) were purchased from Invitrogen-Life Technologies (Carlsbad, CA, USA). The antibodies against PARP (#9542) and anti-rabbit (#7074) secondary antibodies were from Cell Signaling Technology (Beverly, MA, USA). The human apoptosis antibody array was purchased from Abcam (ab134001; Cambridge, MA, USA). Mn (III)-tetrakis (4-benzoic acid)porphyrin chloride (MnTBAP) was purchased from Calbiochem (San Diego, CA, USA).

### 4.2. Quercetin Derivatives: 4Me-Q and 4Ac-Q

4Me-Q (purity > 99%) was purchased from LKT Laboratories (St. Paul, MN, USA). 4Ac-Q was synthesized as described previously [21,22]. For 4Ac-Q synthesis, acetic anhydride (5 equiv.) was dropped into the solution of quercetin (5 mmol) in anhydrous pyridine (6 mL) at room temperature. The mixture was stirred at room temperature for 15 min and then poured into ice-cold water. The precipitate was separated by filtration and then washed with ice-cold water. 4Ac-Q was finally recrystallized from methanol. The chemical structure of acetylated quercetin was determined by ^1^H-NMR, which was recorded on a spectrometer (JEOL ECA-600, 600 MHz for ^1^H, JEOL, Tokyo, Japan) using [D6]CDCl3 as solvents and tetramethylsilane (TMS) as the internal standard. The purity of 4Ac-Q tested, as determined by HPLC, was approximately >98%. A stock solution of quercetin and its derivatives were prepared in dimethyl sulfoxide (DMSO) and diluted with complete media immediately before use. An equal volume of DMSO was added to the controls. ^1^H-NMR (CDCl3) for 4Ae-Q: 2.34 (s, 9H, COCH_3_), 2.38 (s, 3H, COCH_3_), 6.60 (d, J = 6.0 Hz, 1H, aromatic H), 6.85 (d, J = 6.0 Hz, 1H, aromatic H), 7.36 (d, J = 12 Hz, 1H, aromatic H), and 7.74 (d, J = 12 Hz, 1H, aromatic H).

### 4.3. Cell Culture

An MCF-7 cell line was purchased from American Type Culture Collection (Manassas, VA, USA) and cultured in Eagle’s Minimum Essential Medium (EMEN) supplemented with 0.01 mg/mL human recombinant insulin, 1% NEAA, 1% sodium pyruvate, and 10% fetal bovine serum (FBS) in a 5% CO_2_-containing atmosphere. MDA-MB-231 cell lines which were kindly provided by Dr. Shivendra V. Singh (UPMC Hillman Cancer Center, Pittsburgh, PA, USA) and were maintained in high glucose-containing RPMI1640 medium at 37 °C in a humidified atmosphere of 5% CO_2_. RPMI-1640 was supplemented with 10% FBS, 2 mM L-glutamine, and 1% penicillin–streptomycin–neomycin (PSN) antibiotic mixture.

### 4.4. Cell Viability Assay

Cell viability was performed by an MTS assay. MCF-7 (3.0 × 10^3^/well) or MDA-MB-231 cells (4.0 × 10^3^/well) were placed in a 96-well plate overnight and incubated with quercetin, 4Me-Q, and 4Ac-Q at the final concentration (20, 40, 80, and 160 μM) for 48 h. We added 15 µL of CellTiter 96^®^ AQueous One Solution Reagent per well. We incubated the plate at 37 °C for 4 h in a 5% CO_2_ atmosphere. We recorded the absorbance at 490 nm using a 96-well plate reader. The cell viability was expressed as the optical density ratio of the treatment to control.

### 4.5. Colony Formation Assay

MCF-7 and MDA-MB-231 cells were collected by centrifugation. Then, resuspended cells were seeded in a 6-well plate at a density of 5000 cells/well of MCF-7 cells and MDA-MB-231 cells in 2 mL of medium. After 24 h of incubation, the culture medium was replaced by a fresh medium containing 10 or 20 μM of each compound. Ten days later, both cell clones were stained with a solution containing 0.5% crystal violet and were counted using Image J 1.50i software (Wayne Rasband, KY, USA).

### 4.6. Detection of Apoptosis through Annexin V-FITC/Propidium Iodide Flow Cytometry

For the quantitation of apoptosis by flow cytometry using an annexin V-FITC/propidium iodide kit, cells were treated with 0.1% DMSO or quercetin, 4Me-Q, and 4Ac-Q for 48 h. Cells were harvested and washed with phosphate-buffered saline (PBS). Cells were suspended in 100 mL of binding buffer and stained with 4 μL of annexin V-FITC and 2 μL of propidium iodide solution for 15 min at room temperature in the dark. Samples were then diluted with 200 μL of binding buffer. Stained cells were analyzed using a Coulter Epics XL flow cytometer.

### 4.7. Detection of Apoptosis through the Measurement of Cytoplasmic Histone-Related DNA Fragments

The quantitation of histone-associated DNA fragment release into the cytosol was performed according to the manufacturer’s instructions using the Cell Death Detection ELISA^PLUS^ kit from Roche Applied Sciences (Roche Diagnostics, Basel, Switzerland). MCF-7 cells (3.0 × 10^4^ cells/well) or MDA-MB-231 cells (4.8 × 10^4^ cells/well) were seeded in 12-well plates and treated with the respective samples. After cell lysis and centrifugation, the supernatant was transferred to an ELISA plate and incubated with an incubation buffer containing anti-histone biotin and anti-DNA POD for 2 h with shaking. Following washing with incubation buffer, 100 μL of ABTS substrate buffer was added, and absorbance was measured at 405 and 490 nm using a Multiskan^TM^ FC spectrophotometer (Thermo Scientific^TM^, Waltham, MA, USA). The optical density ratio of the treatment to the control was used to calculate the apoptosis induction enrichment factor.

### 4.8. Western Blot Analysis

The Western blot analysis procedure closely followed previously established protocols [56]. Briefly, cells were collected and lysed using RIPA buffer, which consisted of 50 mM Tris-HCl (pH 8.0), 150 mM NaCl, 1 mM EDTA, 1% Nonidet P-40, 0.25% Na-deoxycholate, 1 mM sodium fluoride, 1 mM sodium orthovanadate, 1 mM phenylmethylsulfhonyl fluoride, and a proteinase inhibitor cocktail (Nacalai Tesque, Kyoto, Japan). The equal amounts of lysate protein were run on SDS-PAGE and electrophoretically transferred onto a PVDF membrane (GE Healthcare UK, Amersham, Buckinghamshire, UK). After blocking, membranes underwent overnight incubation with specific primary antibodies at 4 °C, followed by exposure to corresponding HRP-conjugated secondary antibodies for 1 h at room temperature. The visualization of protein bands was achieved using the ECL system.

### 4.9. Determination of ROS Production and Mitochondrial Membrane Potential

ROS production was measured by flow cytometry with the use of a chemical probe (MitoSOX red). MCF-7 and MDA-MB-231 cells were treated with DMSO (control) or 40 μM quercetin, 4Me-Q, and 4Ac-Q for 30 min and then incubated with 5 mM MitoSOX red for 30 min. Cells were collected and washed with PBS, and fluorescence was detected using a flow cytometer. The mitochondrial membrane potential in the DMSO-treated control and samples-treated cells was determined by flow cytometry using JC-1 essentially as previously described by us [22].

### 4.10. Trypan Blue Assay with MnTBAP Co-Treatment Cells

MCF-7 and MDA-MB-231 cells were pretreated with 50 μM MnTBAP for 1 h and then co-treated with 40 μM quercetin or 4Ac-Q for another 48 h. The cells were trypsinized and collected by centrifugation. Then, we resuspended cells with trypan blue solution and counted using a TC20 automated cell counter (Bio-Rad, Hercules, CA, USA).

### 4.11. Apoptosis Protein Arrays

The proteome profiling of apoptosis-related proteins was evaluated using the Human Apoptosis Antibody Array—Membrane (43 Targets) (Abcam, Cambridge, UK) according to the manufacturer’s manual. Briefly, MCF-7 and MDA-MB-231 cells were treated with DMSO (control), 40 μM quercetin, or 4Ac-Q, respectively, and then cultured for 48 h. Each cell lysate was prepared with the lysis buffer of the array kit. Cell lysates were diluted and incubated overnight at 4 °C with membranes in which 43 different apoptotic antibodies were arrayed/spotted. Membranes were washed and incubated with a cocktail of biotinylated detection antibodies. Each membrane was then detected using streptavidin HRP-conjugated antibodies and chemiluminescence and was scanned and evaluated using LumiVisionAnalyzer140 software (TAITEC, Saitama, Japan).

### 4.12. Statistical Analysis

Statistical analysis of the data was determined by a one-way analysis of variance (ANOVA) test followed by Tukey’s multiple comparison test, employing GraphPad Prism 9.5.1 software (San Diego, CA, USA). The data are expressed as the means ± standard deviation (SD). All experiments, except for the apoptosis protein arrays, were performed in biological triplicates (*n* = 3) with at least three replicates. For protein arrays, two replicate experiments were performed, and statistics were performed based on all means. The significance level was set as *p*-values of less than 0.05.

## 5. Conclusions

Our study demonstrated that both quercetin and 4Ac-Q induce apoptosis in human breast cancer cells via distinct pathways: p53-dependent in MCF-7 cells and caspase-3-dependent in MDA-MB231 cells. Furthermore, we observed that 4Ac-Q exhibits significantly higher antitumor activity compared with quercetin, particularly in MCF-7 cells, where it triggers apoptosis through a superoxide-independent pathway. These findings present new insights into the anticancer effects of 4Ac-Q and suggest its potential as a method to enhance the efficacy of quercetin in breast cancer.

## Figures and Tables

**Table 1:** Antiproliferative activities of quercetin, 4Me-Q, and 4Ac-Q.

Cell Line		Compound	
	Quercetin	4Me-Q	4Ac-Q
MCF-7	73.16 ± 1.62	>160	36.90 ± 0.98
MDA-MB-231	85.09 ± 1.76	>160	48.30 ± 1.08

IC_50_ ± SD values in μM. After 48 h incubation. IC_50_ values were calculated from dose-response curves.

## Data Availability

The data used to support the findings of this study are available from the corresponding author upon request.
